# Early Intra-Articular Complement Activation in Ankle Fractures

**DOI:** 10.1155/2014/426893

**Published:** 2014-05-21

**Authors:** Hagen Schmal, Gian M. Salzmann, Philipp Niemeyer, Elia Langenmair, Renfeng Guo, Conny Schneider, Maria Habel, Niels Riedemann

**Affiliations:** ^1^Department of Orthopedics and Trauma Surgery, Albert-Ludwigs University Medical Center Freiburg, 79106 Freiburg, Germany; ^2^Inflarx, 07745 Jena, Germany

## Abstract

Cytokine regulation possibly influences long term outcome following ankle fractures, but little is known about synovial fracture biochemistry. Eight patients with an ankle dislocation fracture were included in a prospective case series and matched with patients suffering from grade 2 osteochondritis dissecans (OCD) of the ankle. All fractures needed external fixation during which joint effusions were collected. Fluid analysis was done by ELISA measuring aggrecan, bFGF, IL-1**β**, IGF-1, and the complement components C3a, C5a, and C5b-9. The time periods between occurrence of fracture and collection of effusion were only significantly associated with synovial aggrecan and C5b-9 levels (*P* < 0.001). Furthermore, synovial expressions of both proteins correlated with each other (*P* < 0.001). Although IL-1**β** expression was relatively low, intra-articular levels correlated with C5a (*P* < 0.01) and serological C-reactive protein concentrations 2 days after surgery (*P* < 0.05). Joint effusions were initially dominated by neutrophils, but the portion of monocytes constantly increased reaching 50% at day 6 after fracture (*P* < 0.02). Whereas aggrecan and IL-1**β** concentrations were not different in fracture and OCD patients, bFGF, IGF-1, and all complement components were significantly higher concentrated in ankle joints with fractures (*P* < 0.01). Complement activation and inflammatory cell infiltration characterize the joint biology following acute ankle fractures.

## 1. Introduction

Restoration of anatomy may be sometimes challenging but it is regularly possible in the majority of patients with ankle fractures. However, the risk to develop posttraumatic osteoarthritis (OA) after 10 years is overall almost 40% [1]. Fracture complexity, increasing body mass index, age over 30 years, and length of time since surgery were identified as significant risk factors. This indicates that the understanding of the pathological synovial cytokine regulation within the joint cavity following biochemical or biomechanical impacts may be crucial to develop sufficient interventional strategies accompanying the conventional treatment methods [2]. Since degeneration of cartilage is one of the decisive steps in OA development, dysfunction and disturbed control of mediators influencing cartilage metabolism following different pathologies of the ankle may be hypothesized [3]. A recent analysis of intra-articular cytokine levels following anterior cruciate ligament injury could demonstrate the increased levels of the proinflammatory cytokines IL-6 and IL-8, which might possibly be responsible for triggering cartilage catabolism [4]. Following this concept, administration of intra-articular IL-1 receptor antagonist within the first month following severe knee injury reduced knee pain and improved function over a 2-week interval [5]. Although activation of the complement cascade has been highlighted in different acute diseases as sepsis and rheumatic diseases [6], until now the role of complement in fracture healing and the possible involvement in the development of posttraumatic OA remain undefined. Downstream effects of C5a include upregulation of IL-8 [7] and other proinflammatory mediators, which have been described to trigger catabolic metabolism of cartilage. Furthermore, several animal models suggested that complement plays a significant role in fracture healing, because components were expressed in callus tissue and fracture repair was significantly reduced in C5-deficient mice [8]. This is supported by other* in vitro* and animal studies [9–11].

Aim of this study was the analysis of synovial protein concentrations following ankle dislocation fractures hypothesizing time dependent and specific regulation patterns including activation of complement components.

## 2. Material and Methods

Eight patients were enrolled in a prospective clinical trial between April and September 2013. All suffered from ankle dislocation fractures needing external fixation prior to open reduction. Seven patients underwent additional arthroscopy at time of external fixation. Effusions and lavage fluids were collected and analyzed. For time frame analysis, different patients were sampled at different time points. The first approach would be much stronger than the second. The study was approved by the Ethical Board of the University of Freiburg (AN-EK-FRBRG-335/08) and registered at the German Clinical Trials Register (CORRCYT, DRKS00000365). A written informed consent for participation in the study was obtained from participants or, where participants are children, a parent or guardian.

### 2.1. Specimen Collection

Effusions were collected by anterolateral puncture of the ankle joint prior to arthroscopy and external fixation. The obtained effusions were hemorrhagic with drops of grease; volumes ranged between 1.0 mL and 3.5 mL. Synovial lavage fluids from ankles of patients undergoing an arthroscopy were also intraoperatively collected. Before starting the arthroscopy, 20 mL of sterile ringer solution was instilled into the joint cavity. The fluid was mixed within the joint by repeated passive flexion-extension and repeated manipulation of the posterior and anterior ankle regions and then was aspirated as described before [12]. Obtained volumes ranged between 8 mL and 12.5 mL. Specimens were immediately stored frozen in liquid nitrogen until being analyzed.

### 2.2. Characterization of Patients

In order to compare biochemical regulation patterns between patients with ankle fractures and patients with intact cartilage, all patients with fractures were matched to patients with an osteochondritis dissecans (OCD) grade 2 according to Zwingmann et al. [13]. The patients were selected from a population recently characterized [12]. Criteria for pairwise matching were gender (4 males and 4 females in each group), age (38.9 ± 20.6 years versus 31.8 ± 14.9 years, *P* = 0.409), and body mass index (24.4 ± 2.9 versus. 24.0 ± 4.9, *P* = 0.855). The time between occurrence of fracture and external fixation combined with puncture and/or joint lavage was 41 ± 47 hours. Overall, patients were healthy with an average ASA classification [14] of 1.4 ± 0.7. There was no infection and no open fracture; the average closed soft tissue damage according to Tscherne and Oestern [15] was 1.75 ± 0.5. Furthermore, leucocyte counts and C-reactive protein (CRP) in the patients' serum were recorded prior to index operation and 2 days after surgery. For cell differentiation of effusions, automatic counting in the clinics' hematological department was used. For the technical reason of centralized and automatic counting, specimens used for differential cell counting could not be utilized for measurement of cytokines. Therefore, additional four patients were included in the study (average age 42.3 ± 15.3 years, two male and two female individuals, average BMI 28.3 ± 5.6, no open fractures, no infections, average closed soft tissue damage 1.75 ± 0.5, and average time to puncture 88 ± 60 hours).

### 2.3. Mathematical Analysis of Specimens

Before comparing the concentrations of synovial proteins, different models for extrapolation were analyzed. In case of the patients with ankle fractures, in 7 cases cytokine levels in effusions and lavage fluids could be compared, and different methods for calculation of absolute concentrations were tested. The most reliable method was the multiplication of the measured concentration in the lavage fluids with a correction factor resulting from the proportion of the mean total protein concentrations of effusion and joint flushes. Calculations with relations of individual concentrations of an examined cytokine and total protein content either followed by correction or not diminished the quality of correspondence with real concentrations which were known from the measurements of effusions. Furthermore, the conclusion for comparisons of cytokine relations is different from the comparison of absolute concentrations. The following criteria were applied for quality control: statistical significance for difference of diluted and absolute concentrations, no difference of corrected concentrations, and statistical significance of correlation analysis. Since the same method for lavaging the joint was applied in fracture and OCD ankle joints, the same correction factor was assumed and absolute concentrations of cytokines were compared.

### 2.4. ELISAs for Aggrecan, bFGF, IL-1*β*, IGF-1, and BCA (Bicinchoninic Acid) Protein Assay

In order to measure concentrations of the indicated proteins, commercially available ELISA kits provided by R&D Systems (Wiesbaden-Nordenstadt, Germany) for bFGF, IGF-1, and IL-1*β* and BioSource (BioSource Deutschland GmbH, Solingen, Germany) for aggrecan were used according to the manufacturers' instructions. Briefly, the assay employed the quantitative sandwich enzyme immunoassay technique. A specific monoclonal antibody (MAb) was precoated onto a microplate. Supernatants were applied to the wells and, after washing, an HRP-conjugated specific Ab was added to the wells. Following the next wash, color development was proportional to protein concentration and was calculated by comparison with a standard. Measurements were done in duplicates, and high stability and reproducibility were assured by measuring the same samples at different time points with concordant results. This confirmed the reliability of the storing procedure as well as the ELISA method as also reported by the manufacturers. Furthermore, standard controls always had standard deviations less than 5%. A colorimetric method was used in order to quantify total protein amount in lavage fluids and effusions. The bicinchoninic acid (BCA) assay was available in kit form from Pierce (Rockford, IL, USA) and was used according to the manufacturers' instructions. Measurements were done in quadruplicates.

### 2.5. ELISAs for C3a, C5a, and C5b-9

In order to measure C3a, C5a, and C5b-9 concentrations, commercial ELISA kits from BD Bioscience were used and measured in the InflaRx Laboratory (Jena, Germany). C3a and C5a ELISAs are “ready-to-use” kits and are characterized by BD Bioscience. These ELISAs were performed according to the manufacturers' instructions. The C5b-9 ELISA was validated by the InflaRx Laboratory based on the ELISA set (including coating and detection antibody and C5b-9 standard) provided by BD Bioscience. The C3a, C5a, and C5b-9 ELISAs were used for human biological samples with the reference values in activated plasma from healthy humans of 530 ng/mL for C3a, 15 ng/mL for C5a, and 74 ng/mL for C5b-9.

### 2.6. Statistical Analysis

All values were expressed as mean ± standard deviation. Normal probability plots were done on all data sets and correlation was determined by either calculating the Pearson (*R*) or the Spearmen (*ρ*) coefficient depending on distribution. This was complemented by a regression plot including the 95% confidence intervals (CI). Individual group means of protein or cytokine concentrations were compared with the unpaired Student's* t*-test; in case of unequal variances, the Aspin-Welch test has been applied. In case of not normally distributed values or scores, individual group means were compared with the Mann-Whitney* U* rank sum test. Statistical significance was defined when *P* < 0.05.

## 3. Results

### 3.1. Correlation of Synovial Cytokine Levels with Fracture Characterizing Parameters

Age and body mass index (BMI) did not statistically significantly correlate with any of the analyzed cytokines or their relative expressions (cytokine/total protein content (TPC)). General health status was evaluated using the ASA classification and no statistically significant correlation was found except for total C3a (*ρ* = 0.65, *P* = 0.04), but not for the relative expression. The time periods between occurrence of fracture and collection of effusion were highly significantly associated with total synovial aggrecan (*ρ* = 0.74, *P* = 0.018), relative aggrecan expression (*ρ* = 0.81, *P* = 0.0007), and relative C5b-9 levels (*ρ* = 0.81, *P* = 0.0007, Table 1). Furthermore, synovial expressions of both proteins correlated with each other (*ρ* = 0.74, *P* = 0.018). The other investigated proteins did not show time dependent statistically significant expressions. Although IL-1*β* expression was relatively low (average 101.6 ± 134.6 pg/mL), intra-articular levels correlated with C5a (*ρ* = 0.83, *P* = 0.0051) and serological CRP concentrations 2 days after surgery (*ρ* = 0.64, *P* = 0.043).

### 3.2. Comparison of Intra-Articular Concentrations of Cytokines in Patients with Fracture or OCD Grade 2

**Figure 1:**
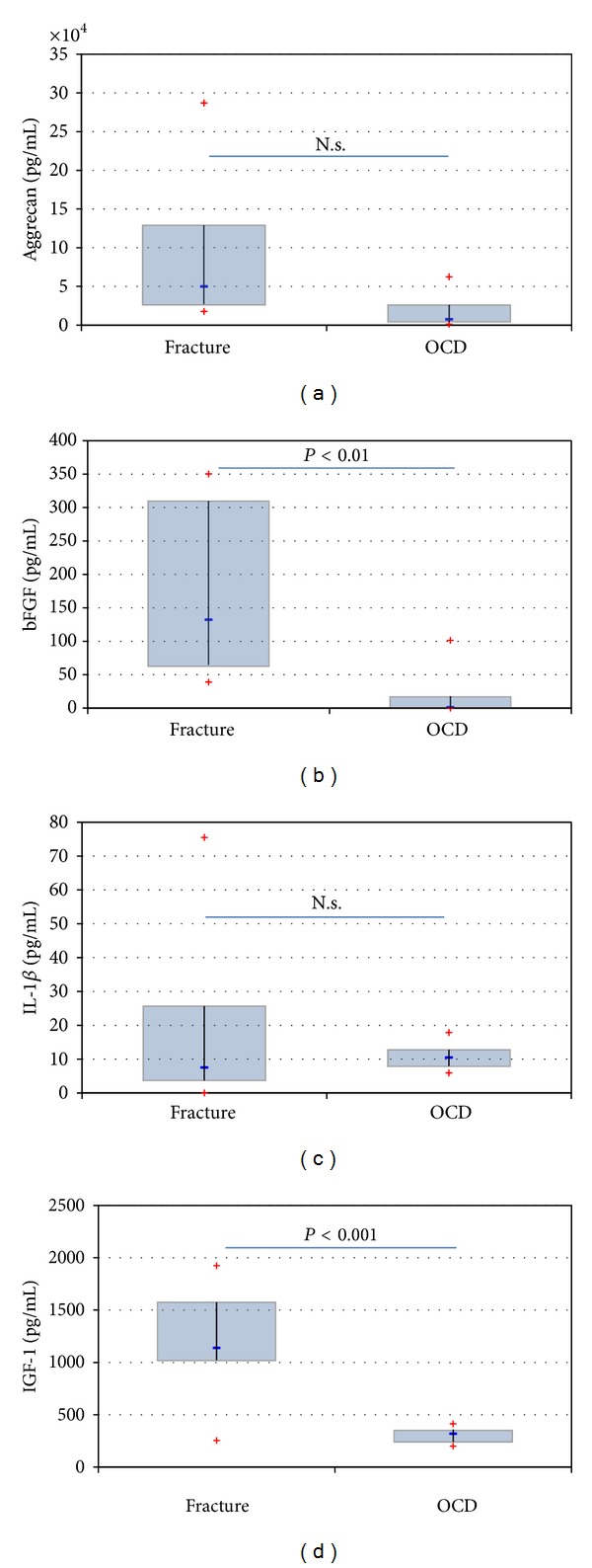
Showing the comparison of different synovial levels in patients with ankle dislocation fractures and patients suffering from an OCD grade 2. There was no statistically significant difference for aggrecan (a) and IL-1*β* (c). The difference reaches statistical significance in the case of bFGF ((b) *P* = 0.0007) and IGF-1 ((d) *P* = 0.0013). N.s.: not significant.

**Figure 2:**
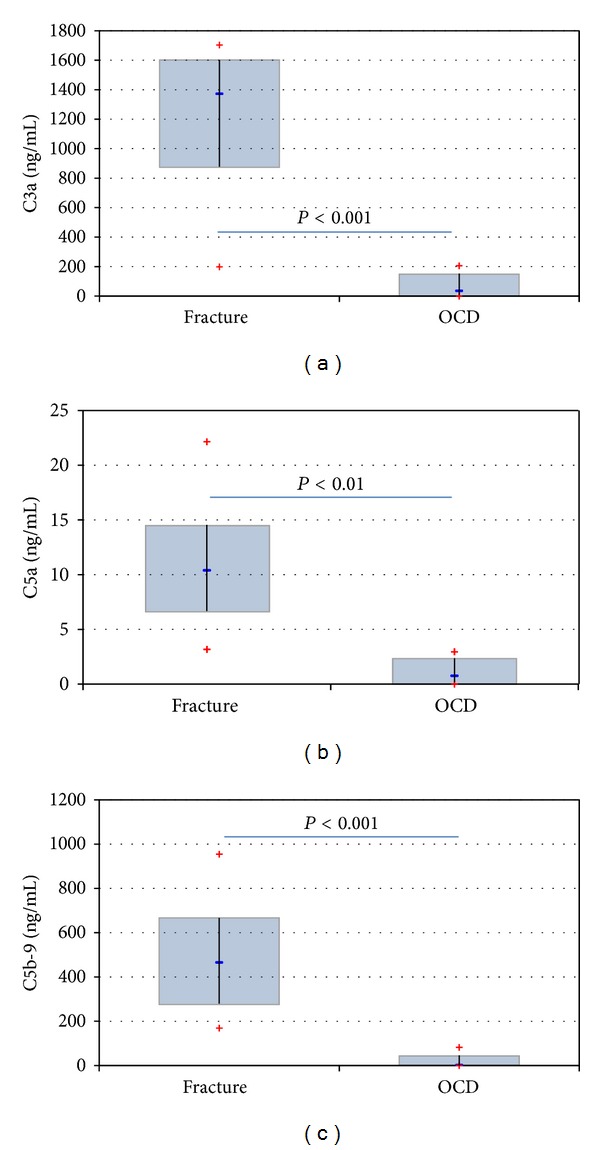
Showing the comparison of C3a levels ((a) *P* = 0.0003), C5a levels ((b) *P* = 0.0018) and C5b-9 levels ((c) *P* = 0.0013) in patients with ankle dislocation fractures and patients suffering from an OCD grade 2. The difference was statistically significant for all comparisons.

The cytokine concentrations were compared using the corrected absolute values in effusions (*n* = 8) and the levels were directly measured in the joint lavages (*n* = 7). The indicated concentrations report only the corrected absolute values that also were used in the figures, but* P* values were provided for both comparisons in order to support the statistical evidence. Whereas aggrecan (85,243 ± 91,512 pg/mL versus 16,842 ± 20,443 pg/mL, *P* = 0.074 for corrected values, *P* = 0.230 for flushes) and IL-1*β* (18.7 ± 24.8 pg/mL versus 10.9 ± 3.7 pg/mL, *P* = 0.40 for corrected values, *P* = 0.396 for flushes) concentrations were not different in fracture and OCD patients (Figures 1(a) and 1(c)), bFGF (173.0 ± 120.4 pg/mL versus 16.7 ± 35.0 pg/mL, *P* = 0.0007 for corrected values shown in Figure 1(b), *P* = 0.047 for flushes), IGF-1 (1198 ± 493 pg/mL versus 307 ± 73 pg/mL, *P* = 0.0013 for corrected values shown in Figure 1(d), *P* = 0.0029 for flushes), and all complement components (C3a 1218 ± 506 pg/mL versus 73 ± 78 pg/mL, *P* = 0.0003 for corrected values shown in Figure 2(a), *P* = 0.007 for flushes, C5a 11 ± 5.9 pg/mL versus 1.1 ± 1.2 pg/mL, *P* = 0.0018 for corrected values shown in Figure 2(b), *P* = 0.0036 for flushes, C5b-9 484 ± 256 pg/mL versus 21.1 ± 31.2 pg/mL, *P* = 0.0013 for corrected values shown in Figure 2(c), *P* = 0.03 for flushes) were significantly higher concentrated in ankle joints with fractures.

### 3.3. Analysis of Cell Composition in Effusions of Ankle Joint after Dislocation Fracture

**Figure 3:**
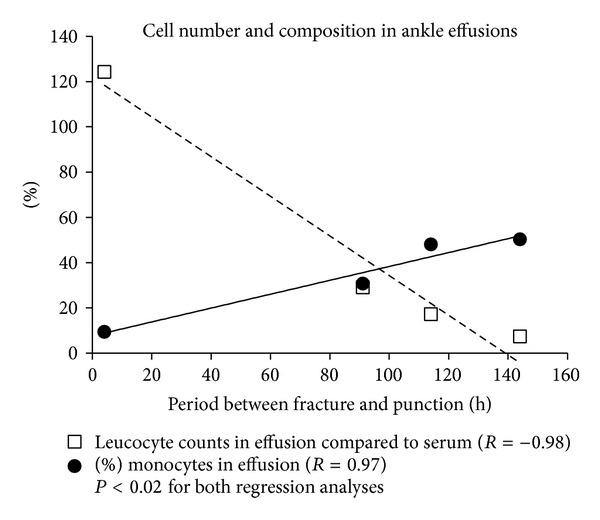
The relation of leucocytes in effusion and leucocytes in blood decreased over time showing a statistically significant correlation (*R* = −0.98, *P* = 0.011, dashed line). In parallel, the percentage of monocytes in joint flushes increased over time starting with 9.5% 4 hours after trauma (90.5% neutrophils), reaching 50.4% 144 hours (49.6% neutrophils) following injury (*R* = 0.97, *P* = 0.013, solid line).

Neither cell counts for leucocytes (8.5 ± 0.81 thousand cells/µL) nor cell counts for erythrocytes (4.0 ± 0.96 thousand cells/µL) in blood did show significant differences comparing the four different patients. In contrast, the composition of the effusions was dependent on the time period between occurrence of fracture and time point of puncture. At first the total number of leucocytes decreased over time; therefore, the relation of leucocytes in effusion and leucocytes in blood decreased over time showing a statistically significant correlation (*R* = −0.98, *P* = 0.011, Figure 3). In parallel, the percentage of monocytes increased over time starting with 9.5% 4 hours after trauma (90.5% neutrophils found for the same time point), reaching 50.4% 144 hours (49.6% neutrophils found for the same time point) following injury (*R* = 0.97, *P* = 0.013, Figure 3). At no time point did the proportion of leucocytes and erythrocytes in blood equal the same proportion in the effusions.

## 4. Discussion

The main finding of the study was the identification of complement activation following acute ankle fractures, determining an initial inflammatory cytokine milieu. Within the first 6 days following occurrence of fracture, the synovial levels of aggrecan and C5b-9 are increasing, indicating degradation of extracellular cartilage matrix (ECM) and accumulation of end-products of complement activation as part of the fracture specific biochemical regulation. The analysis of the cellular level of joint effusions following ankle dislocation fractures could demonstrate that the composition of blood and synovial fluid is completely different at any examined time point and that a shift from neutrophils to monocytes characterizes the first days after injury within the joint.

Aggrecan is an essential and specific proteoglycan of the ECM, which contributes to the effective neutralization of compressive forces. Aggrecanase mediated degradation of aggrecan is known to play a significant role in the development of osteoarthritis (OA) [16]. Correlating, proteoglycan fragments were identified as markers for progress of OA [17]. This was supported by a study correlating the radiological OA-related changes of ankles and loss of function with increased intra-articular aggrecan concentrations [12]. However, the simple presence of circumscribed cartilage lesions was not suitable to induce elevated aggrecan release in knees [18], suggesting a dynamic component of synovial aggrecan regulation in association with OA development. The time factor may also explain the lacking difference between synovial levels in ankles with fracture or OCD, because the increase of aggrecan concentrations following fractures happens over time. Furthermore, the OCD itself may cause some ECM degradation as well. There is no doubt that a fracture may be a starting point for OA [1], which is supported by the presented data indicating the association of synovial posttraumatic biology with OA-typical changes as elevation of aggrecan levels. Whether this may be initiated by impact-related chondrocyte death as recently suggested [19] may not be concluded by the data of this clinical trial. Examination of sheared off cartilage fragments gained during open reduction of these fractures may possibly clarify this in the future.

There is growing evidence that fractures and high mechanical impacts resulting in soft tissue injuries are accompanied with and followed by inflammatory-like reactions, defining the first inflammatory phase of the repair process followed by the actual repair and the remodeling phases [20]. Although this has been known for years, the debate about the key mediators in this process is ongoing. Recently, the proinflammatory cytokines IL-6 and IL-8 were found to be upregulated after anterior cruciate ligament (ACL) injury [4]. Another group pointed out the importance of IL-1*β*, administering intra-articular IL-1 receptor antagonist (IL-1Ra) in order to improve the outcome following ACL tear [5]. Although the number of the study populations was limited, effects seen for function and swelling were encouraging. Our data indicate the intra-articular presence and a certain role for IL-1*β* within the joint cavity following ankle fracture, because measured concentrations correlated with synovial C5a and serological CRP, but the levels did not differ from patients with an early stage of OCD. This population was chosen because it may ethically not be justified to obtain samples from healthy individuals without need for operative revision and because this disease is not supposed to be associated with a relevant accompanying inflammatory reaction. Furthermore, OCD stage 2 is defined by an undisturbed cartilage layer, identifying these joints as healthy as possible for a comparison with the fracture joints. The measured low level expression of IL-1*β* confirmed this assumption; however, concentrations were still higher compared to levels found in healthy knee joints [18]. As described in the knee, the association between synovial IL-1*β* expressions with serological CRP concentrations 2 days later could be confirmed for the ankle [18]. Since this limited expression of the proinflammatory key cytokine IL-1*β* may barely explain the observed downstream effects as the neutrophil influx, complement components known to be involved in a variety of acute and chronic inflammatory diseases [21] were measured and found to be elevated. One possible link between complement activation and osteoblast/osteoclast functioning is supposed to be C5a with activating and chemotactic activity for these cells [9–11]. Mesenchymal stem cells, expressing C5a and TNF*α* receptors [22], are supposed to play a significant role in fracture healing as well [23]. Although TNF*α* was not addressed in the presented study and synovial elevation of this cytokine following ACL rupture could not be demonstrated [4], TNF*α* was able to increase C3a receptor expression in cultured articular, auricular, and nasoseptal chondrocytes [24]. In the same* in vitro* study, it was shown that the majority of the investigated complement factors as C3a and C5a and the complement regulatory proteins CD35, CD46, CD55, and CD59 were expressed at a significantly lower level in nonarticular chondrocytes compared to articular chondrocytes. These* in vitro* data are in line with our* in vivo* findings showing high intra-articular complement activation including C5a expression in the very first phase following ankle dislocation fractures. Although these cellular and biochemical mechanisms may be suspected, the understanding of the complete effects of synovial complement activation following fracture requires further investigations. It is not yet clear how complement is activated following fractures, but one may speculate that activation is probably driven by binding of cell debris to C1q, activating the classical or the lectin pathway [25, 26]. Also the alternative pathway has been shown to be activated on the surface of apoptotic cells [27]. The fracture may further cause some molecules that are normally hidden within the ECM to be exposed to complement components in the synovial fluid [28]. This may further activate complement and toll-like receptors, which may enhance an already existing inflammatory response. One only may speculate whether complement activation as an initial event of inflammation is good or bad for the necessary repair processes. Probably the truth is somewhere in between, because the chemotactic activity for osteoblasts and osteoclasts as well as for neutrophils supports fracture healing and removal of cell debris. But the consequences also include possible cartilage destruction. Since the ability of bone to heal is better than cartilage partial inhibition of complement activation, this may be beneficial for the long term joint prognosis.

An inflammatory-like biology within the joint cavity following fractures may also be assumed analyzing the data collected about cell-type regulation. Within the first hours after fracture the intra-articular milieu is predominated by neutrophils as described for other complement driven diseases as sepsis [29]. The fraction of monocytes including macrophages as osteoclasts and stem cells grows over the next days, reaching a portion of more than 50% of the leucocytes at day 6. This is in line with recently published data from an animal model supporting an important role for MCP-1 in the early phase of stress fracture repair and activated remodeling [30]. Moreover, the specific cell patterns, which are different from blood at all time points, indicate a specific joint biology.

Not only complement factors but also bFGF and IGF-1 were statistically significantly elevated in ankles following fractures compared to OCD. Both proteins are known to play an important role in cartilage metabolism and were upregulated in knees with focal cartilage defects compared to healthy individuals [18]. Osteoarthritic progression is thought to be associated with the upregulation of bFGF, which also may be expected in posttraumatic degeneration [31]. This is supported by the fact that cartilage injury leads to release of bFGF from the chondrocytes themselves [32]. bFGF increases chondrocyte proliferation and has controversial effects on differentiation, leading to the conclusion that bFGF is necessary for a functional balance during repair processes [32]. bFGF has been shown to inhibit the anabolic effect of IGF-1 [33], a cytokine with immanent importance as a promoter of growth and matrix synthesis by chondrocytes in healthy articular cartilage. IGF-1 enhances aggrecan synthesis by articular cartilage cells or explants, which has been demonstrated in cell culture experiments and using* in vivo* animal models [34]. In this context, increased bFGF and IGF-1 expression in ankles following dislocation fractures indicate a direct impact on cartilage resulting in the induction of intrinsic repair processes.

A weak point of the study is the limited number of included patients. The publication may be justified by the clear differences seen in cytokine regulation between the groups compared including the statistical significance. Furthermore, confirming effects were observed as aggregation of complement end-products over time similar to aggrecan and cytokine-specific regulation patterns. It should be noted that the ELISAs for C3a and C5a also measure their desarginated forms, C3a-desArg and C5a-desArg. These may be the predominant forms of these factors present within the joint cavity, and functional assays were not performed.

In summary, complement activation and cartilage matrix degradation could be shown within the first days after trauma following dislocation fractures of the ankle. Both phenomena appear to contribute to the genesis of posttraumatic OA and offer possible interventional opportunities.

## Figures and Tables

**Table 1:** Correlation of intra-articular protein levels with age, serum CRP at second day after surgery, BMI, ASA classification, and period between fracture and punction [h]. Statistical tests refer to correlations to the absolute protein concentrations and the protein concentrations in relation to TPC. Data indicate degradation of extracellular matrix of cartilage and activation of complement over time following fracture. Spearman's *ρ* and *P* values are only provided in case of statistical significance; in these cases, all values ranged within the 95% CI.

	Age	Postoperative CRP	BMI	ASA	Period fracture/punction [h]
TPC					
Corr. coefficient	—	**−0.64**	—	—	—
Valid cases	8	**8**	8	8	8
Signif. (*P*)	N.s.	**0.0428**	n.s.	N.s.	N.s.
Aggrecan/ Aggrecan/TPC					
Corr. coefficient	—/—	—/—	—/—	—/—	**0.74/0.81**
Valid cases	8	8	8	8	**8**
Signif. (*P*)	N.s./n.s.	N.s./n.s.	N.s./n.s.	N.s./n.s.	**0.001/0.005**
bFGF/bFGF/TPC					
Corr. coefficient	—/—	—/—	—/—	—/—	—/—
Valid cases	8	8	8	8	8
Signif. (*P*)	N.s./n.s.	N.s./n.s.	N.s./n.s.	N.s./n.s.	N.s./n.s.
IL-1*β*/IL-1*β*/TPC					
Corr. coefficient	—/—	—**/0.64**	—/—	—/—	—/—
Valid cases	8	**8**	8	8	8
Signif. (P)	N.s./n.s.	**n.s./0.0428**	N.s./n.s.	N.s./n.s.	N.s./n.s.
IGF-1/IGF-1/TPC					
Corr. coefficient	—/—	—/—	—/—	—/—	—/—
Valid cases	8	8	8	8	8
Signif. (*P*)	N.s./n.s.	N.s./n.s.	N.s./n.s.	N.s./n.s.	N.s./n.s.
C3a/C3a/TPC					
Corr. coefficient	—/—	—/—	—/—	**0.65/—**	—/—
Valid cases	8	8	8	**8**	8
Signif. (*P*)	N.s./n.s.	N.s./n.s.	N.s./n.s.	**0.0391/n.s.**	N.s./n.s.
C5a/C5a/TPC					
Corr. coefficient	—/—	—/—	—/—	—/—	—/—
Valid cases	8	8	8	8	8
Signif. (*P*)	N.s./n.s.	N.s./n.s.	N.s./n.s.	N.s./n.s.	N.s./n.s.
C5b-9/C5b-9/TPC					
Corr. coefficient	—/—	—/—	—/—	—/—	**—/0.81**
Valid cases	8	8	8	8	**8**
Signif. (*P*)	N.s./n.s.	N.s./n.s.	N.s./n.s.	N.s./n.s.	**N.s./0.007**

BMI: body mass index; ASA: Physical Status Classification System of the American Society of Anesthesiologists; TPC: total protein content; CI: confidence interval; signif. (*P*): *P* value for statistical significance; Corr. coefficient: correlation coefficient (Spearman's *ρ*); N.s.: not significant.

## References

[1] Lubbeke A, Salvo D, Stern R, Hoffmeyer P, Holzer N, Assal M (2012). Risk factors for post-traumatic osteoarthritis of the ankle: an eighteen year follow-up study. *International Orthopaedics*.

[2] Kramer WC, Hendricks KJ, Wang J (2011). Pathogenetic mechanisms of posttraumatic osteoarthritis: opportunities for early intervention. *International Journal of Clinical and Experimental Medicine*.

[3] Patra D, Sandell LJ (2011). Recent advances in biomarkers in osteoarthritis. *Current Opinion in Rheumatology*.

[4] Bigoni M, Sacerdote P, Turati M (2013). Acute and late changes in intraarticular cytokine levels following anterior cruciate ligament injury. *Journal of Orthopaedic Research*.

[5] Kraus VB, Birmingham J, Stabler TV (2012). Effects of intraarticular IL1-Ra for acute anterior cruciate ligament knee injury: a randomized controlled pilot trial (NCT00332254). *Osteoarthritis and Cartilage*.

[6] Czermak BJ, Sarma V, Pierson CL (1999). Protective effects of C5a blockade in sepsis. *Nature Medicine*.

[7] Wang L, Han G, Wang R (2010). Regulation of IL-8 production by complement-activated product, C5a, in vitro and in vivo during sepsis. *Clinical Immunology*.

[8] Ehrnthaller C, Huber-Lang M, Nilsson P (2013). Complement c3 and c5 deficiency affects fracture healing. *PLoS ONE*.

[9] Hengartner NE, Fiedler J, Ignatius A, Brenner RE (2013). IL-1beta inhibits human osteoblast migration. *Molecular Medicine*.

[10] Ignatius A, Ehrnthaller C, Brenner RE (2011). The anaphylatoxin receptor C5aR is present during fracture healing in rats and mediates osteoblast migration in vitro. *Journal of Trauma: Injury, Infection and Critical Care*.

[11] Ignatius A, Schoengraf P, Kreja L (2011). Complement C3a and C5a modulate osteoclast formation and inflammatory response of osteoblasts in synergism with IL-1*β*. *Journal of Cellular Biochemistry*.

[12] Schmal H, Henkelmann R, Mehlhorn AT (2013). Synovial cytokine expression in ankle osteoarthritis depends on age and stage. *Knee Surgery Sports Traumatology Arthroscopy*.

[13] Zwingmann J, Sudkamp NP, Schmal H, Niemeyer P (2012). Surgical treatment of osteochondritis dissecans of the talus: a systematic review. *Archives of Orthopaedic and Trauma Surgery*.

[14] Keats AS (1978). The ASA classification of physical status—a recapitulation. *Anesthesiology*.

[15] Tscherne H, Oestern HJ (1982). A new classification of soft-tissue damage in open and closed fractures. *Traumatology*.

[16] Huang K, Wu LD (2010). Suppression of aggrecanase: a novel protective mechanism of dehydroepiandrosterone in osteoarthritis→. *Molecular Biology Reports*.

[17] Lohmander LS, Hoerrner LA, Lark MW (1993). Metalloproteinases, tissue inhibitor, and proteoglycan fragments in knee synovial fluid in human osteoarthritis. *Arthritis and Rheumatism*.

[18] Schmal H, Mehlhorn A, Stoffel F, Kstler W, Sdkamp NP, Niemeyer P (2009). In vivo quantification of intraarticular cytokines in knees during natural and surgically induced cartilage repair. *Cytotherapy*.

[19] Jang KW, Buckwalter JA, Martin JA (2013). Inhibition of cell-matrix adhesions prevents cartilage chondrocyte death following impact injury. *Journal of Orthopaedic Research*.

[20] Claes L, Recknagel S, Ignatius A (2012). Fracture healing under healthy and inflammatory conditions. *Nature Reviews Rheumatology*.

[21] Guo RF, Ward PA (2005). Role of C5a in inflammatory responses. *Annual Review of Immunology*.

[22] Schmal H, Niemeyer P, Roesslein M (2007). Comparison of cellular functionality of human mesenchymal stromal cells and PBMC. *Cytotherapy*.

[23] Song K, Rao NJ, Chen ML, Huang ZJ, Cao YG (2011). Enhanced bone regeneration with sequential delivery of basic fibroblast growth factor and sonic hedgehog. *Injury*.

[24] Schulze-Tanzil G, Kohl B, El Sayed K (2012). Anaphylatoxin receptors and complement regulatory proteins in human articular and non-articular chondrocytes: interrelation with cytokines. *Cell and Tissue Research*.

[25] Korb LC, Ahearn JM (1997). C1q binds directly and specifically to surface blebs of apoptotic human keratinocytes: complement deficiency and systemic lupus erythematosus revisited. *The Journal of Immunology*.

[26] Ogden CA, DeCathelineau A, Hoffmann PR (2001). C1q and mannose binding lectin engagement of cell surface calreticulin and CD91 initiates macropinocytosis and uptake of apoptotic cells. *Journal of Experimental Medicine*.

[27] Nagasawa S (1994). Activation of the alternative pathway of complement by apoptotic Jurkat cells. *FEBS Letters*.

[28] Happonen KE, Heinegard D, Saxne T, Blom AM (2012). Interactions of the complement system with molecules of extracellular matrix: relevance for joint diseases. *Immunobiology*.

[29] Riedemann NC, Guo RF, Gao H (2004). Regulatory role of C5a on macrophage migration inhibitory factor release from neutrophils. *Journal of Immunology*.

[30] Wu AC, Morrison NA, Kelly WL, Forwood MR (2013). MCP-1 expression is specifically regulated during activation of skeletal repair and remodeling. *Calcified Tissue International*.

[31] Vincent T, Hermansson M, Bolton M, Wait R, Saklatvala J (2002). Basic FGF mediates an immediate response of articular cartilage to mechanical injury. *Proceedings of the National Academy of Sciences of the United States of America*.

[32] Schmal H, Zwingmann J, Fehrenbach M (2007). bFGF influences human articular chondrocyte differentiation. *Cytotherapy*.

[33] Loeser RF, Chubinskaya S, Pacione C, Im HJ (2005). Basic fibroblast growth factor inhibits the anabolic activity of insulin-like growth factor 1 and osteogenic protein 1 in adult human articular chondrocytes. *Arthritis and Rheumatism*.

[34] Loeser RF, Pacione CA, Chubinskaya S (2003). The combination of insulin-like growth factor 1 and osteogenic protein 1 promotes increased survival of and matrix synthesis by normal and osteoarthritic human articular chondrocytes. *Arthritis and Rheumatism*.

